# Plastid genomics in horticultural species: importance and applications for plant population genetics, evolution, and biotechnology

**DOI:** 10.3389/fpls.2015.00586

**Published:** 2015-07-30

**Authors:** Marcelo Rogalski, Leila do Nascimento Vieira, Hugo P. Fraga, Miguel P. Guerra

**Affiliations:** ^1^Laboratório de Fisiologia Molecular de Plantas, Departamento de Biologia Vegetal, Universidade Federal de ViçosaViçosa, Brazil; ^2^Laboratório de Fisiologia do Desenvolvimento e Genética Vegetal, Programa de Pós-graduação em Recursos Genéticos Vegetais, Centro de Ciências Agrárias, Universidade Federal de Santa CatarinaFlorianópolis, Brazil

**Keywords:** plastome, horticultural crops, conservation, photosynthesis, plastid genetic engineering

## Abstract

During the evolution of the eukaryotic cell, plastids, and mitochondria arose from an endosymbiotic process, which determined the presence of three genetic compartments into the incipient plant cell. After that, these three genetic materials from host and symbiont suffered several rearrangements, bringing on a complex interaction between nuclear and organellar gene products. Nowadays, plastids harbor a small genome with ∼130 genes in a 100–220 kb sequence in higher plants. Plastid genes are mostly highly conserved between plant species, being useful for phylogenetic analysis in higher taxa. However, intergenic spacers have a relatively higher mutation rate and are important markers to phylogeographical and plant population genetics analyses. The predominant uniparental inheritance of plastids is like a highly desirable feature for phylogeny studies. Moreover, the gene content and genome rearrangements are efficient tools to capture and understand evolutionary events between different plant species. Currently, genetic engineering of the plastid genome (plastome) offers a number of attractive advantages as high-level of foreign protein expression, marker gene excision, gene expression in operon and transgene containment because of maternal inheritance of plastid genome in most crops. Therefore, plastid genome can be used for adding new characteristics related to synthesis of metabolic compounds, biopharmaceutical, and tolerance to biotic and abiotic stresses. Here, we describe the importance and applications of plastid genome as tools for genetic and evolutionary studies, and plastid transformation focusing on increasing the performance of horticultural species in the field.

## Introduction

The existence of plastids represents one of the principal features that distinguish plant from other eukaryotic cells. Except for some gametic cells, plastids are assumed to be present as one of several different types in all living cells of higher plants, which show its essentiality for cell viability (Zhang et al., 2003; Kuroiwa, 2010; Nagata, 2010). These different plastid types have specific characteristics and functions, i.e., proplastids (present in meristematic regions of the plant); chloroplasts (chlorophyll-containg plastids specialized in photosynthesis); chromoplasts (colored plastids able to store high amounts of carotenoids present in petals of flowers and fruits); amyloplasts (mainly present in storage tissues such as tubers and seed endosperm); elaioplasts (lipid-storing plastids); leucoplasts (pigment-less plastids present mainly in root cells); and etioplasts (achlorophyllous plastids present in cotyledons of dark-grown angiosperm seedlings; Lopez-Juez and Pyke, 2005; Egea et al., 2010; Pyke, 2011; Bock, 2014; Osteryoung and Pyke, 2014). Moreover, plastids are involved in other essential cellular processes such as lipid, hormone, amino acid, and phytochrome biosynthesis as well as nitrate and sulfate assimilation (Tetlow et al., 2004; Waters et al., 2004; Aldridge et al., 2005; Rogalski and Carrer, 2011; Galili and Amir, 2013; Galili et al., 2014).

**FIGURE 1:**
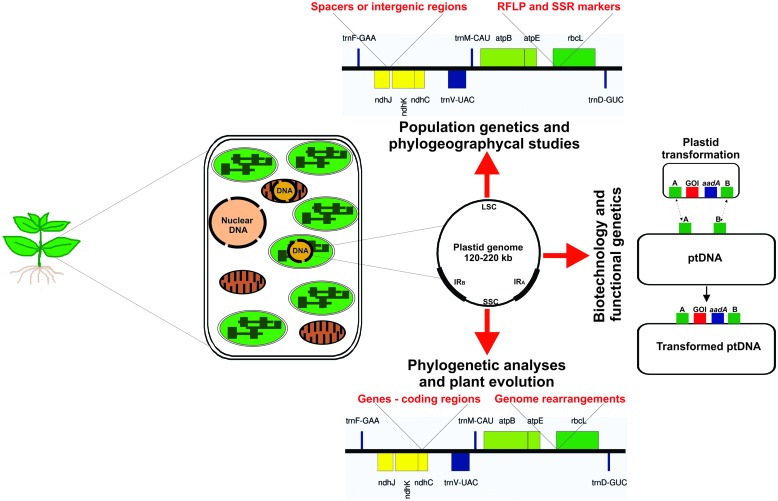
**Illustration of a plant cell shows the genetic material into the three cellular compartments.** Different sequences of plastid DNA are used for several applications as population genetics and phylogeographycal studies (intergenic spacers, RFLP and SSR molecular markers), plant biotechnology (intergenic spacers used as targeted position for integration of transgenes), functional genetics of plastid genes (the mutated allele is inserted into the functional gene revealing the gene function) and mechanisms involved in the plastid gene expression machinery (mutation in genes involved in plastid genome transcription and translation elucidating the processes), and for phylogenetic and evolutionary analyses (use of whole plastid genome or coding region to determine the evolutionary history of plant groups, e.g., family, genus, and at species level). ptDNA – plastid DNA.

At the beginning of the last century, a non-Mendelian inheritance of leaf variegation in *Mirabilis jalapa* and *Pelargonium zonale* was proposed, suggesting the plastids would contain their own genome (Baur, 1909, 1910; Correns, 1909; Hagemann, 2000, 2002; Greiner et al., 2011). This hypothesis was confirmed with the discovery of plastid DNA (Chun et al., 1963; Sager and Ishida, 1963; Tewari and Wildman, 1966). Today we know that the plastid genome (plastome) size of photosynthetically active seed plants varies between 120 and 220 kb in a circularly mapping genome (**Figure 1**), encoding 120–130 genes. The plastome is commonly mapped as a single circular molecule, however, it shows a high dynamic structure (i.e., linear molecules, branched complexes, and circular molecules) and ploydy level in each chloroplast (Bendich, 2004). Thus, inside a single cell, the plastome may occur at high copy number, with up to thousands of genome copies. Mesophyll cells of higher plants can contain 700–2000 copies of plastome, which depend on the developmental stage of the leaves and the plant species (Golczyk et al., 2014). These multiple copies are packed together in large nucleoprotein bodies, the plastid nucleoids (Golczyk et al., 2014; Krupinska et al., 2014; Powikrowska et al., 2014). Generally, the plastid DNA in photosynthetic active plant tissues (i.e., chloroplasts) forms up to 10–20% of total cellular DNA content (Bendich, 1987; Bock, 2001; Golczyk et al., 2014).

Although the evolutionary forces that gave rise to the characteristic diversity of sizes, rearrangements, structure, and compactness of contemporary plastomes are poorly understood, nowadays the plastome has been used as basis for analyses of phylogeny and evolution (Leebens-Mack et al., 2005; Jansen et al., 2007; Parks et al., 2009; Moore et al., 2010; Crosby and Smith, 2012; Vieira et al., 2014a), population genetics (Angioi et al., 2009b; Nock et al., 2011; Yang et al., 2013; Wheeler et al., 2014), plastid gene transfer to nucleus (Huang et al., 2003, 2005; Stegemann et al., 2003; Bock, 2006; Stegemann and Bock, 2006), exchange of plastome between different species (Stegemann et al., 2012; Fuentes et al., 2014), plant speciation (Greiner et al., 2011), functional genomics (Svab et al., 1990; Svab and Maliga, 1993), and plastid gene expression machinery (Ruf et al., 1997; Hager et al., 1999; Drescher et al., 2000; Shikanai et al., 2001; Maliga, 2004; Kode et al., 2005; Rogalski et al., 2006, 2008; Alkatib et al., 2012). In addition to basic research, plastome studies may be focused in plastome transformation for biotechnological applications, i.e., adding new agronomic traits, manipulation of metabolic pathways, enhanced pest resistance, increase of biomass and production of enzyme for biofuel industry, and molecular farming in species related to agriculture and horticulture (Maliga and Bock, 2011; Verma et al., 2013; Wang et al., 2013; Bock, 2014; Shenoy et al., 2014; Shil et al., 2014; Zhang et al., 2015). All of these plastid/plastome applications are summarized in the **Figure 1**.

Here, we review recent progress in plastid genomics in horticultural species. We focus on plastid evolution, gene content, size, inheritance, genomic structure, and rearrangements. We present information about the plastid genome of horticultural species and the current use of this information for different areas. We also briefly highlight the application the plastid genome information on genetic diversity and divergence within natural plant populations, evolution and the importance of plastid genomic for biotechnological use.

## Plastid Origin and Evolution

The evolutionary history of plastids is based on the endosymbiotic theory which posits that plastids and mitochondria originated from an engulfment of free-living eubacteria over a billion years ago, an α-proteobacteria and a cyanobacterium ancestor, respectively, giving rise the present-day plant cell (Timmis et al., 2004; Reyes-Prieto et al., 2007; Bock and Timmis, 2008; Gould et al., 2008; Archibald, 2009; Kleine et al., 2009; Keeling, 2013; Zimorski et al., 2014). The main evidence of the origin of organelles via the endosymbiotic theory is the molecular, genetic, physiological, and biochemical similarities to prokaryotic cells of ancestors (Zimorski et al., 2014). From these symbionts, the eukaryotic cell acquired the novel biochemistry as oxidative phosphorylation and photosynthesis (Timmis et al., 2004; Bock and Timmis, 2008; Green, 2011). The acquisition of organelles was one of the most important evolutionary processes, given that the association between host and symbionts resulted in a cell with three compartments containing genetic information: the nucleus, mitochondria, and plastids. The combination of three genomes, or host and symbiont genetic compartments, was followed by a dramatic reorganization of the genomes with loss of dispensable genes from organelles, elimination of common genetic information, transfer of genes from organelles to the nucleus, import of products of these transferred genes into the organelles and a complex interaction between nuclear and organellar gene products with the acquisition of new gene functions (Martin et al., 1998; Dyall et al., 2004; Timmis et al., 2004; Bock, 2006; Bock and Timmis, 2008; Zimorski et al., 2014). As a consequence, the size of organelle genomes was drastically reduced during the evolution of the plant cell (Bock, 2007, 2014; Jansen and Ruhlman, 2012).

Nowadays, plastids retain a small prokaryotic chromosome containing no more than 200 protein coding genes (Glöckner et al., 2000; Zimorski et al., 2014) from more than 3200 present in their cyanobacterial ancestor (Kaneko et al., 1996). Even containing a reduced genome and small number of protein-coding genes, plastids harbor thousands of proteins (Meisinger et al., 2008; Kleine et al., 2009), which means that the plastid proteome do not reflect its genome. Most of the genes present into the symbiont genome reside now in the nucleus, where they became functional and their products (i.e., proteins) continue to have their original function in the plastids. Some genes have similarly migrated to the nucleus, however, acquiring a new function which is not related to the prokaryotic ancestor (Martin et al., 2002; Rousseau-Gueutin et al., 2012). Proteomic and genomic analyses suggest that approximately 93–99% of the proteins present in plastids are encoded in the nucleus (Richly et al., 2003; Richly and Leister, 2004; Meisinger et al., 2008; Kleine et al., 2009).

Some experiments *in vivo* were carried out to recapitulate the movement of plastid DNA to the nucleus by using of tobacco transplastomic plants (Huang et al., 2003, 2005; Stegemann et al., 2003; Bock and Timmis, 2008). These experiments suggest that, during the evolution, organellar DNA have been constantly transferred to the nucleus and regularly incorporated into chromosomes. Few experiments have been done to show how a plastid gene becomes functional in the nucleus (Stegemann and Bock, 2006) and the stability of gene expression after nuclear insertion (Sheppard and Timmis, 2009). These different experimental approaches using transplastomic plants showed that the gene transfer from plastid genome to the nucleus is an ongoing process and occurs at a surprisingly high frequency (Huang et al., 2003, 2005; Stegemann et al., 2003; Bock, 2006; Stegemann and Bock, 2006; Bock and Timmis, 2008).

Following the reduction of plastome size, gene content and expression capacity during the evolution, some angiosperm species acquired new lifestyle as parasite plants as examples *Epifagus virginiana* and different species of the *Cuscuta* genus (Wolfe et al., 1992; Lohan and Wolfe, 1998; McNeal et al., 2007). This adaptation to the new life has resulted in an attenuation of the plastome and plastid gene expression machinery and, consequently, a high dependency on the host plant. These alterations in the plastome include loss of photosynthesis-related genes, deletion of a meaningful part of the genetic information and impaired photosynthesis capacity in some species (Funk et al., 2007; Gould et al., 2008; Tsai and Manos, 2010). Except in parasite plants where photosynthesis is dispensable, the plastome sequence and gene content of different species of higher plants are highly conserved, and some experimental evidences *in vivo* have suggested that plastid gene expression is essential for cell survival and development (Rogalski et al., 2006, 2008; Alkatib et al., 2012; Tiller and Bock, 2014). However, a different situation was observed in the high-throughput sequencing and transcriptomic analyses of *Polytomella* spp., a free-living non-photosynthetic green algae closely related to the model organism *Chlamydomonas reinhardtii* (Smith and Lee, 2014). For this species, data analyses revealed no plastid genome-derived reads and, although *Polytomella* spp. has plastids, they do not contain a genome and seems to be only a cellular compartment needed for cellular metabolism (Smith and Lee, 2014).

## Regulation of Gene Expression in Plastids

Plastid gene expression involves not only the activation of a set of plastid genes required for plastid biogenesis and photosynthesis, but also the modulation of gene expression during chloroplast development and in response to different environmental factors (Pfannschmidt, 2003; Pfannschmidt et al., 2003). The plastid gene expression must rely on the nucleus for most of their structural proteins and regulatory factors, and a complex signaling pathways are involved, demonstrating the interdependence and need for coordination of gene expression between these cellular genetic compartments (Bräutigam et al., 2007; Greiner et al., 2011). All steps of plastid gene expression are dependent on nuclear gene expression given that nuclear gene products (i.e., proteins) are required for transcription, processing, translation, post-translation modification, and turnover of plastid proteins (Marín-Navarro et al., 2007; Wobbe et al., 2008; Berry et al., 2013; Chi et al., 2013; Small et al., 2013; Petrillo et al., 2014; Ramundo and Rochaix, 2014).

This complex interaction between nuclear genome and organellar genome (i.e., plastome) plays a crucial role in the plant cell controlling the entire metabolism. Moreover, organellar and nuclear genomes constitute a tightly integrated functional unit that co-evolves. This integration between cellular genomes (e.g., plastid genome and nuclear genome) is involved in speciation processes where the lack of functional interaction between genomes results in reproductive barriers between populations (Greiner et al., 2011). Fail in the interaction between plastid genome and nuclear genome can induce genome incompatibilities affecting phenotypically the progenies and resulting in hybrid bleaching, hybrid variegation, or disturbance of the sexual phase (Bogdanova, 2007; Greiner et al., 2008, 2015; Bogdanova et al., 2009; Greiner and Bock, 2013), which affect directly the survival of the plants on natural environment. The sequencing of plastid genome, because of its small size and relatively low number of genes, is a valuable and essential tool to investigate the cause of these incompatibilities (Greiner et al., 2008, 2011; Besnard et al., 2011; Bogdanova et al., 2012; Greiner and Bock, 2013).

## Plant Population Genetic Studies in Horticultural Species based on Plastid Genomes

Plastid genomes, unlike most nuclear chromosomes, are typically uniparentally inherited. For sexually reproducing species with male and female gametes, maternal plastid inheritance is the norm (Zhang and Sodmergen, 2010), although it was indicated that about 20% of angiosperms exhibits the potential for paternal plastid transmission (Corriveau and Coleman, 1988; Zhang et al., 2003; Zhang and Sodmergen, 2010; Schneider et al., 2015). Studies have identified diverse species with paternal (mainly conifers) or biparental modes of plastid inheritance (Crosby and Smith, 2012). This uniparental mode of inheritance allows the generation of inferences about the relative contributions of seed and pollen flow to the genetic structure of natural populations by comparing nuclear and plastid markers (Provan et al., 2001; Roullier et al., 2011; Delplancke et al., 2012; Khadivi-Khub et al., 2013).

Effective genetic population size is a parameter influenced by the mode of inheritance. The haploid nature of chloroplast genome is related to its reduced genetic variation. Since the effective population size of a haploid genome is 1/4 in dioecious plants and 1/2 in monoecious plants of the nuclear genome, coalescence times and time to fixation of chloroplast DNA haplotypes within a population are shorter than in diploid genomes (Small et al., 2004). Moreover, different plastid genes evolve at different rates, allowing measuring evolutionary distance at many taxonomy levels (Palmer, 1985; Shaw et al., 2007). This low evolving rate along with the absence of recombination, uniparentally inherited nature in most plant species perceived in plastid genome may greatly facilitate the use of plastid DNA markers in plant population genetic studies (Palmer, 1985; Powell et al., 1995; Provan et al., 2001).

In the 80’s, the use of phylogenetic studies based on plastid genomes began to show promising results (Palmer, 1985). The *rbcL* gene was widely sequenced from many plant taxa, generating a suitable database for plant phylogenetic studies at family level and higher taxa (Palmer et al., 1988; Hasebe et al., 1992; Brunsfeld et al., 1994; Setoguchi et al., 1998). However, in some cases *rbcL* gene and other coding regions proved to be highly conserved searching the answer of questions between closely related genera (Gielly and Taberlet, 1994). Since the non-coding regions are likely to evolve faster than coding regions (Gielly and Taberlet, 1994), the analysis of non-coding regions of plastid DNA (i.e., introns and intergenic spacers) was a strategy applied to clarify the relationships at lower taxonomic levels, see **Figure 1**. This strategy has solved some of the questions in the context of phylogenetic studies, and later many unexplored non-coding regions of the plastid genome proved promising to bring even more additional information to this line of study (Shaw et al., 2005, 2007). For instance, the pairwise sequence divergence across genes, introns, and spacers in *Helianthus* (Asteraceae) and *Lactuca* (Asteraceae) has resulted in the discovery of fast-evolving DNA sequences for use in species-level phylogenetics (Timme et al., 2007).

The plastid markers, restriction fragment length polymorphisms (RFLPs), began to be used for evolution (Palmer et al., 1983), phylogenetic (Jansen and Palmer, 1987; Sandbrink et al., 1989), and plastid diversity analyses (Ogihara and Tsunewaki, 1988; Dally and Second, 1990). This method has the disadvantage of requiring reasonable amounts plant material, digestible, and nuclear DNA-free plastid DNA, associated with laborious experimental procedures of the Southern hybridization-based RFLP method (Sandbrink et al., 1989; Provan et al., 2001).

In the following decade, Powell et al. (1995) reported the presence of simple nucleotide repeats in plastids, exhibiting length variation and polymorphism in higher levels than those of plastid RFLPs (**Figure 1**). This marker became a widely used plastid marker, known as chloroplast simple sequence repeats (cpSSRs), consisting of repetitive DNA sequences in tandemly repeated motifs of six base pairs (bp) or less, which have aroused considerable interest due to their ability to generate highly informative DNA markers (Provan et al., 2001). Even though in chloroplasts genomes the occurrence of di-, tri-, tetra-, penta-, and hexanucleotide repeats is less common (George et al., 2015). These regions may be used for both intraspecific and interspecific variability analyses, with practical value for monitoring gene flow, population differentiation and cytoplasmic diversity (Powell et al., 1995).

The development and application of these plastid molecular markers was demonstrated by Angioi et al. (2009a), who developed a useful set of cpSSR markers to study the genetic diversity of *Phaseolus* spp. and other legumes. These markers could discriminate among the genera, and among and within the species of the *Phaseolus* genus. Shortly after, these set of markers were applied to characterized a *Phaseolus vulgaris* collection from Italy, clarifying the origin of the Sardinian (Italy) bean germplasm by comparing local accessions with commercial and Americas varieties (Angioi et al., 2009b). These data generated important information to elucidate the colonization process of *P. vulgaris* in Europe and to define an appropriate management of the local genetic resources, particularly for breeding purposes.

Similarly, Khadivi-Khub et al. (2013) characterized the genetic and phylogenetic relationships of eight wild *Prunus L.* subgen. C*erasus* species naturally growing in Iran and three commercial species based on nuclear and cpSSR. These markers were able to discriminate all species analyzed, with high level of polymorphism detected, indicating high inter-and intraspecific genetic variation. A close correlation was observed between intraspecific variation and geographical distribution, providing bases for conservation suggestion for these native populations of wild *Cerasus* germplasm and for future breeding activity. By using the same strategy of combining nuclear and cpSSR markers, but for different purposes, Delplancke et al. (2012) investigated spontaneous gene flow among wild and domesticated *Prunus*. Two key almond tree species were selected, the cultivated *Prunus dulcis* and the wild relative *Prunus orientalis*. They identified high genetic diversity levels in both species along with substantial and symmetric gene flow between the domesticated and the wild species. This crop-to-wild gene flow study highlights the importance of use of *ad hoc* transgene containment strategies for this species before the introduction of genetically modified cultivars.

The cpSSRs can also be applied to elucidate evolutionary questions in high economic interesting species and with intriguing domestication processes, such as the evolutionary history of wheat species. The characterization of a large set of accessions of *Triticum spp.*, provided very strong evidence that neither *Triticum urartu* nor *Aegilops tauschii* was the maternal and thus cytoplasmic donor for polyploid wheats cultivated today (Leigh et al., 2013).

In sweet potato landraces, nuclear and cpSSR markers combined also allowed the origin and dispersal investigation, providing bases to suggest at least two independent domestications processes for these species, in Central/Caribbean America and in the north-western part of South America. The comparison of nuclear and chloroplast data also suggests that exchanges of clones and sexual reproduction were both important processes in landrace diversification in this clonally propagated crop. These analyses provided useful tools for rationalizing the conservation and use of sweet potato germplasm collections (Roullier et al., 2011).

Another relevant area of cpDNA markers application is phylogeographical analysis, e.g., in following colonization after the ice age. The fact that chloroplasts present mainly uniparental inheritance means that they show a clearer geographical structure than nuclear markers, notably in wind-pollinated species (Petit et al., 2005). In this way, glacial refugia have been identified for several tree species, such as *Quercus petraea, Quercus pubescens*, *Fagus sylvatica* (Petit et al., 2002), and *Populus nigra* (Cottrell et al., 2005).

## Plastid Genome in Horticultural Species

In general, land plant chloroplast genomes are mostly conserved and contain basically two groups of genes. The first group comprises components for the photosynthetic machinery – photosystem I (PSI), photosystem II (PSII), the cytochrome b6/f complex and the ATP synthase. The second group includes the genes required for the genetic system of plastids – subunits of an RNA polymerase, rRNAs, tRNAs, and ribosomal proteins. The tobacco plastid genome, for example, consists of 155,943 bp and contains a pair of inverted repeat regions (IRA and IRB) separated by a small (SSC) and a large (LSC) single copy region (**Figure 1**). It contains 115 genes, 79 protein-encoding genes and 35 encoding stable RNA species (Shinozaki et al., 1986; Wakasugi et al., 1998; Yukawa et al., 2005). This plastid genome organization is highly conserved in angiosperms, with very few exceptions (Guo et al., 2007; Hansen et al., 2007; Tangphatsornruang et al., 2010; Do et al., 2014; Gurdon and Maliga, 2014). In gymnosperms, the loss of the large IR has been reported in several species, mainly in conifers (**Table 1**; Hirao et al., 2008; Wu and Chaw, 2013; Yi et al., 2013; Vieira et al., 2014a). Some authors believed that a pair of large IR could stabilize the plastid genome against major structural rearrangements (Palmer and Thompson, 1982; Strauss et al., 1988; Hirao et al., 2008). However, recently Sabir et al. (2014) found evidences that the loss of the IR in legumes is not the major driving force behind the genomic upheaval, and hypothesized that other factors, such as the extent and location of repetitive DNA, may be more important in destabilizing these genomes.

**Table 1:** List of genes category, group, and names commonly identified in plant plastid genomes.

Category of genes	Group of gene	Name of gene					
**Self-replication**	Ribosomal RNA genes	*rrn16*	*rrn23*	*rrn5*	*rrn4.5*		
	Transfer RNA genes	*trnA* -UGC	*trnC* -GCA	*trnD* -GUC	*trnE* -UUC	*trnF* -GAA	*trnfM* -CAU
		*trnG* -UCC	*trnG* -GCC	*trnH* -GUG	*trnI* -CAU	*trnI* -GAU	*trnK* -UUU
		*trnL* -CAA	*trnL* -UAA	*trnL* -UAG	*trnM* -CAU	*trnN* -GUU	*trnP* -GGG
		*trnP* -UGG	*trnQ* -UUG	*trnR* -ACG	*trnR* -UCU	*trnR* -CCG	*trnS* -GCU
		*trnS* -UGA	*trnS* -GGA	*trnT* -UGU	*trnT* -GGU	*trnV* -GAC	*trnV* -UAC
		*trnW* -CCA	*trnY* -GUA				
	Small subunit of ribosome	*rps2*	*rps3*	*rps4*	*rps7*	*rps8*	*rps11*
		*rps12*	*rps14*	*rps15*	*rps16*	*rps18*	*rps19*
	Large subunit of ribosome	*rpl2* *rpl32*	*rpl14* *rpl33*	*rpl16* *rpl36*	*rpl20*	*rpl22*	*rpl23*
	DNA-dependent RNA polymerase	*rpoA*	*rpoB*	*rpoC1*	*rpoC2*		
	Translational initiation factor	*infA*					
**Genes for photosynthesis**	Subunits of photosystem I (PSI)	*psaA* *ycf3*	*psaB* *ycf4*	*psaC*	*psaI*	*psaJ*	*psaM*
	Subunits of photosystem II (PSII)	*psbA*	*psbB*	*psbC*	*psbD*	*psbE*	*psbF*
		*psbH*	*psbI*	*psbJ*	*psbK*	*psbL*	*psbM*
		*psbN*	*psbT*	*psbZ*			
	Subunits of cytochrome	*petA*	*petB*	*petD*	*petG*	*petL*	*petN*
	Subunits of ATP synthase	*atpA*	*atpB*	*atpE*	*atpF*	*atpH*	*atpI*
	Large subunit of Rubisco	*rbcl*					
	Chlorophyll biosynthesis	*chlB*	*chlL*	*chlN*			
	Subunits of NADH dehydrogenase	*ndhA*	*ndhB*	*ndhC*	*ndhD*	*ndhE*	*ndhF*
		*ndhG*	*ndhH*	*ndhI*	*ndhJ*	*ndhK*	
**Other genes**	Maturase Envelope membrane protein Subunit of acetyl-CoA C-type cytochrome synthesis gene Protease Component of TIC complex	*matK* *cemA* *accD* *ccsA* *clpP* *ycf1*					
**Genes of unknown function**	Conserved open reading frames	*ycf2*	*ycf12*				
**Pseudogenes**		*ycf68*	*ycf15*				

*We used four phylogenetically distant species to unify in a single table the gene content of plastid genomes. Small changes may occur according to species. The species used as reference were Prunus persica (eudicotyledon), Elaeis guineensis (monocotyledon), Picea abies (conifer Clade I), and Taxus mairei (Conifer Clade II)*.

In order to unify in a single table a list of genes category, group, and names commonly identified in plant plastid genomes, we arbitrarily choose four representative horticultural species from very distant taxa, two angiosperms: *Prunus persica* (eudicotyledon) and *Elaeis guineensis* (monocotyledon), and two gymnosperms: *Picea abies* (conifer Clade I) and *Taxus mairei* (Conifer Clade II; **Table 1**). Although plastid genome shows a high conservative gene content, small changes may occur according to the species, such as complete gene losses, or presence of pseudogenes.

The beginning of the complete plastid genome sequencing was in the 1980s, with the almost simultaneously sequence release of *Nicotiana tabacum* (Shinozaki et al., 1986) and *Marchantia polymorpha* (Ohyama et al., 1986). The plastid genome sequencing, especially in tobacco, together with the development of plastid transformation for this species have allowed the investigation of the function of several plastid genes (Svab et al., 1990; Svab and Maliga, 1993). Consequently, during the next years until the present, plastid genes and gene expression machinery have been extensively studied (Ruf et al., 1997; Hager et al., 1999; Drescher et al., 2000; Shikanai et al., 2001; Maliga, 2004; Kode et al., 2005; Rogalski et al., 2006, 2008; Alkatib et al., 2012).

In the last decades, several research groups around the world have centered its efforts on the sequencing of plastid genomes of various taxonomic groups. Today, more than 600 land plant species has its plastid genome sequence available in Genbank web page (www.ncbi.nlm.nih.gov/genomes/GenomesGroup.cgi?taxid=2759&opt=plastid). In this review, we highlight species of high interest to horticulture, as tomato (NC_007898; Daniell et al., 2006), potato (NC_008096; Chung et al., 2006), lettuce (NC_007578; Timme et al., 2007), spinach (NC_002202; Schmitz-Linneweber et al., 2001), onion (NC_024813), carrot (NC_008325; Ruhlman et al., 2006); ornamental species, as orchids, i.e., *Phalaenopsis aphrodite* (NC_007499; Chang et al., 2006), *Cymbidium aloifolium* (NC_021429; Yang et al., 2013), and *Cattleya crispata* (NC_026568; da Rocha Perini et al., 2015), *Lilium* (NC_026787), *Magnolia kwangsiensis* (NC_015892; Kuang et al., 2011); fruit crops, as strawberry (NC_015206; Shulaev et al., 2011), peach (NC_014697; Jansen et al., 2011), orange (NC_008334; Bausher et al., 2006), banana (HF677508; Martin et al., 2013); medicinal species, as *Camellia grandibracteata* (NC_024659; Huang et al., 2014), *Salvia* (NC_020431; Qian et al., 2013), *Artemisia frigida* (NC_020607; Liu et al., 2013); and forestry species, as *Eucalyptus aromaphloia* (NC_022396; Bayly et al., 2013), *Pinus contorta* (NC_011153; Cronn et al., 2008), *Picea abies* (NC_021456; Nystedt et al., 2013).

The beginning of plastid genome sequencing involved cloning of chloroplast DNA into plasmid vectors, followed by selection of chloroplast DNA-containing clones, and then sequencing the clones in traditional Sanger-based sequencers using both plasmid and chloroplast-specific primers (Jansen et al., 2005). With the emergence of pyrosequencing, more specifically with the Genome Sequencer 20 (GS 20) system (Roche, Basel, Switzerland), to clone template DNA into bacterial vectors became no more necessary, and genome sequence could be obtained in a single 5-h run with a few days of template preparation (Moore et al., 2006).

Shortly after, Cronn et al. (2008) PCR-amplified eight *Pinus* plastid genomes and adapted multiplex sequencing-by-synthesis (MSBS) to simultaneously sequence multiple plastid genomes using the Illumina Genome Analyzer (Illumina Inc., San Diego, CA, USA). The use of the PCR-based methods to amplify overlapping fragments from conserved gene loci in plastid genomes is time consuming and can be more difficult to implement considering that gene organization differs among plants (Atherton et al., 2010). Atherton et al. (2010) demonstrated a suitable alternative approach, isolating chloroplasts and then using the capacity of high-throughput sequencer Illumina Genome Analyzer II to obtain purified and complete plastid sequences. This technique allowed the obtainment of reads sequence easy to assemble for building the complete plastid genome map.

With the advances of next-generation sequencing, it is becoming increasingly faster and cost-effective to sequence and assemble plastid genomes. The isolation of chloroplast DNA is a facilitator in the sequencing data assembly (Vieira et al., 2014b), but the capacity of current sequencing technologies allows effective analysis of the chloroplast genome sequence by sequencing total DNA (Henry et al., 2014). Using this approach, the chloroplast insertions in the nuclear genome can be distinguished by their much lower copy number, and the short-read sequences from plastid genome are easy discriminated from nuclear reads by alignment with a reference plastome (Henry et al., 2014). Thus, depending on the available framework, nowadays plastid genome sequence may be realized from amplification of chloroplast DNA using long range PCR in species that chloroplast isolation is more challenged and hard to be reached.

Thereby, the complete genome sequencing in Fabaceae family allowed the comparison in two horticultural species of high economic potential, *Glycine max* and *P. vulgaris* with the considered outstanding model for genome research *Medicago truncatula.* All the three legumes present very similar gene content and order, and lack the *rpl22* gene (Saski et al., 2005; Guo et al., 2007). However, the *rps16* is an intron-containing and functional gene in *G. max*, a pseudogene in *P. vulgaris* and absent in *M. truncatula* (Saski et al., 2005; Guo et al., 2007). *M. truncatula* also differ by missing one copy of the IR (Saski et al., 2005). Studies point out that the presence of small repeats of *psbA* and *rbcL* in legumes that have lost one copy of the IR indicate that this loss has only occurred once during the evolutionary history of legumes (Cai et al., 2008; Gurdon and Maliga, 2014). *P. vulgaris* differs from the others by containing an additional pseudogene, *rpl33*. Interestingly, *P. vulgaris* chloroplast genome show higher evolutionary rates on genomic and gene levels than *G. max*, which is believed to be a consequence of pressure from both mutation and natural selection (Guo et al., 2007).

In Rosids, a large monophyletic clade of Angiosperms, comprising 17 orders, many of them containing species with high economic interest, several plastid genomes were sequenced (Hu et al., 2011; Jansen et al., 2011; Rodríguez-Moreno et al., 2011; Njuguna et al., 2013). These plastid genome sequences enabled the identification of a common gene lost in Passifloraceae and Fagaceae, the *rpl22* (Jansen et al., 2011). In *Passiflora sp.*, *Castaneae* sp., and *Quercus sp.* the *rpl22* was present in the chloroplast genome as a pseudogene, and in *Castanea* sp. and *Quercus* sp. it was identified a complete copy of this gene in the nuclear genome, characterizing a functional gene transfer from plastid to nucleus (Jansen et al., 2011). As described above, some species from Fabaceae family also lacks *rpl22*. These results together allowed Jansen et al. (2011) to suggest that these *rpl22* gene transfers occurred approximately 56–58, 34–37, and 26–27 Ma for the Fabaceae, Fagaceae, and Passifloraceae, respectively (Jansen et al., 2011).

Comparisons of chloroplast genome organization between *Solanum lycopersicum* and *Solanum bulbocastanum* showed that, at gene order, these genomes are identical, and this conservation extends to more distantly related genera (tobacco and *Atropa*) of Solanaceae (Daniell et al., 2006). These authors also analyzed repeated sequences in Solanaceae chloroplast genomes, revealing 42 groups of repeats shared among various members of the family. In addition, 37 of these 42 repeats are found in all four genomes examined, occurring in the same location, either in genes, introns or within intergenic spacers, suggesting a high level of conservation of repeat structure. In the same way, Chung et al. (2006) reported that the complete sequence of *Solanum tuberosum* chloroplast genome revealed extensive similarity to six Solanaceae species in terms of the gene content and structure, suggesting a common chloroplast evolutionary lineage within Solanaceae.

## Plastid Biotechnology of Horticultural Crops

The plastid genome genetic engineering of crop plants is an attractive platform for biotechnologists to increase characteristics of interest for agriculture and horticulture (Clarke and Daniell, 2011; Maliga and Bock, 2011; Rogalski and Carrer, 2011; Hanson et al., 2013; Bock, 2014). This technology offers several exceptional features and advantages when compared with nuclear transformation, among which can be included high transgene expression levels with accumulation of foreign proteins up to >70% of the total soluble cellular protein (Oey et al., 2009; Ruhlman et al., 2010), capacity for multigene stacking in operons in a single genetic transformation event (Quesada-Vargas et al., 2005; Lu et al., 2013; Bock, 2014), precise transgene integration via homologous recombination (Cerutti et al., 1992), absence of epigenetic effects or gene silencing (Bock, 2001, 2013; Maliga, 2004) and exclusion of transgenes transmission by pollen due to maternal inheritance of plastids in most angiosperms (Daniell, 2007; Ruf et al., 2007; Svab and Maliga, 2007). The plastid transformation vector design and the transgene insertion via two homologous recombination events into the plastid genome are illustrated in the **Figure 1**.

Other desirable, but not exclusive, feature is the possibility of efficient elimination of the selection marker gene via Cre-lox site-specific recombination (Lutz et al., 2006), ϕC31 phage site-specific integrase (Kittiwongwattana et al., 2007), serine recombinase Bxb1(Shao et al., 2014) and/or use of direct repeats for gene excision via homologous recombination (Dufourmantel et al., 2007). This is an exceptional advantage because it allows the production of transgenic plants without the insertion of antibiotic resistance genes, eliminate any possibility of antibiotic resistance gene flow to neighboring crop fields or to crop wild relatives growing near the transgenic crops. Moreover, it permits the recycling of selectable marker genes, which can be reused in a new genetic transformation event in the same transgenic plant (Carrer et al., 1993; Svab and Maliga, 1993; Corneille et al., 2001; Barone et al., 2009; Li et al., 2011).

After the successful plastid transformation of the first higher plant species, *N. tabacum* (Svab et al., 1990; Svab and Maliga, 1993), several aspects of plastid transformation were studied and optimized to increase the potential of transplastomic technology for biotechnological aspects such as crop improvement (Jin et al., 2011, 2012; Lu et al., 2013), herbicide, insect, and diseases resistance (Lutz et al., 2001; Ye et al., 2001; Dufourmantel et al., 2005; Bock, 2007; Kiani et al., 2013; Zhang et al., 2015), abiotic and biotic stresses (Kumar et al., 2004; Jin et al., 2011; Bansal et al., 2012; Chen et al., 2014), metabolic engineering (Hasunuma et al., 2008; Apel and Bock, 2009; Lu et al., 2013), phytoremediation (Yun et al., 2009; Ruiz and Daniell, 2009; Ruiz et al., 2011), bioreactors (Maliga and Bock, 2011; Bock, 2013, 2014), vaccines and biopharmaceuticals (Daniell et al., 2009; Clarke et al., 2013; Kwon et al., 2013), enzymes (Petersen and Bock, 2011; Jin et al., 2012; Verma et al., 2013), biomass and raw material for industry (Verma and Daniell, 2007; Agrawal et al., 2011; Jin et al., 2011; Verma et al., 2013). This research have also focused on plastid gene expression in different plastid types in the same plant (Kahlau and Bock, 2008; Valkov et al., 2009), regulatory elements to be used in different plastid types (i.e., chloroplasts, chromoplasts and amyloplasts; Zhang et al., 2012; Caroca et al., 2013) and foreign protein stability in plastids (Apel et al., 2010; Elghabi et al., 2011; De Marchis et al., 2012).

Although tobacco (*N. tabacum*) is the species transformed with the highest efficiency, commercial use of this technology for cultivar improvement is totally dependent on spread of the technology to plant species of agriculture and horticultural interest. Probably, the wide spread of plastid technology to several horticultural crops is dependent on the development of a highly efficient tissue culture system (via organogenesis or somatic embryogenesis), which is observed in tobacco as model species for plastid transformation (Díaz and Koop, 2014; Maliga and Tungsuchat-Huang, 2014; Staub, 2014). Currently, several horticultural species have been efficiently transformed, including tomato (Ruf et al., 2001; Nugent et al., 2005; Ruf and Bock, 2014), lettuce (Lelivelt et al., 2005; Kanamoto et al., 2006; Ruhlman, 2014), potato (Sidorov et al., 1999; Nguyen et al., 2005; Scotti et al., 2011; Valkov et al., 2014), carrot (Kumar et al., 2004), eggplant (Singh et al., 2010; Bansal and Singh, 2014), cabbage (Liu et al., 2007, 2008; Tseng et al., 2014), cauliflower (Nugent et al., 2006) and sugar beet (De Marchis et al., 2009; De Marchis and Bellucci, 2014). Among the horticultural species mentioned above, lettuce, tomato and potato are the most studied species regarding the gene expression and biotechnological applications; lettuce is a model species for edible leaf chloroplasts (Boyhan and Daniell, 2011; Maldaner et al., 2013; Yabuta et al., 2013), tomato and potato are model species for edible organs as fruits and tubers containing chromoplasts (Wurbs et al., 2007; Apel and Bock, 2009; Lu et al., 2013) and amyloplasts (Valkov et al., 2011; Segretin et al., 2012), respectively.

Lettuce, as a model of edible tissue containing chloroplasts, plastid type with the elevated ploidy and highest gene expression (Kahlau and Bock, 2008; Valkov et al., 2009; Zhang et al., 2012; Caroca et al., 2013; Bock, 2014), is currently the target species for expression of antigens, pharmaceutical proteins and vaccines (Boyhan and Daniell, 2011; Maldaner et al., 2013), and also metabolic engineering (Yabuta et al., 2013). The first example of the use of lettuce plastid genome to produce proteins of pharmaceutical interest was made by Boyhan and Daniell (2011), who observed in old lettuce leaves the accumulation of proinsulin up to 53% of total leaf protein. The same study showed that the accumulation was stable even in senescent and dried lettuce leaves, facilitating their processing and storage in the field. This genetic engineering strategy can reduce significantly the costs and facilitate oral delivery of plant-derived pharmaceutical compounds using edible plant leaves (Boyhan and Daniell, 2011). Recently, another study showed the efficient and stable production of the tetra-epitope peptide antigen from E protein of dengue virus in lettuce transplastomic plants (Maldaner et al., 2013). The tetra-epitope peptide expressed in lettuce plastid genomes shows to be efficient to use as antigen in diagnostic assays demonstrating an overall sensitivity of 71.7% and specificity of 100% (Maldaner et al., 2013). Besides to the pharmaceutical area, lettuce chloroplasts were also used to manipulate the metabolic pathway of the tocochromanol (vitamin E) by expression of the enzymes tocopherol cyclase, γ-tocopherol methyltransferase, or both in an operon (Yabuta et al., 2013). The expression of the different genes, alone or combined, resulted in an increase of total tocochromanol content in transplastomic plants, which indicate that chloroplast genetic engineering can be successful used to improve vitamin E quality and quantity in a plant green edible tissue (Yabuta et al., 2013).

The application of plastid transformation technology in tomato was target to metabolic engineering of plastid pigments. The first successful example showed the feasibility to engineer a nutritionally important metabolic human nutrient in non-green plastids. Apel and Bock (2009) overexpressed the enzyme lycopene β-cyclase from the daffodil (*Narcissus pseudonarcissus*) and observed an increase up to 50% in provitamin A content in tomato fruits (an important antioxidant and essential vitamin for human nutrition), which changed the color from red to orange due to the conversion of lycopene into β-carotene. Another example in tomato chloroplasts and chromoplasts was the increase of tocochromanol, which provides tocopherols and tocotrienols (vitamin E), in a complex and successful transcription and translation strategy of a multigene operon containing three genes related to tocochromanol biosynthesis (Lu et al., 2013). The tomato transplastomic plants showed an increase of up to 10-fold in total tocochromanol accumulation (Lu et al., 2013).

Potato contains edible tubers, which have amyloplasts, plastids related to starch accumulation as the plant energetic reserve. Potato is by far the most important non-cereal source of starch and carbohydrates for human nutrition and is the most consumed species in many countries around the world. The first transplastomic events in potato were obtained by Sidorov et al. (1999) and Nguyen et al. (2005) by expression of the resistant marker gene, *aadA*, and the green fluorescent protein (*Gfp*), however, it was a limited method due to the low transformation frequencies and low transgene expression in tubers of potato transplastomic plants. Later, by optimizing of the selection/regeneration procedure, using of new transformation vectors and new regulatory sequences for transgene expression in leaves and tubers, Valkov et al. (2011) confirmed general differences in expression patterns in the two organs containing different plastids leaves (chloroplasts) and tubers (amyloplasts). Although expression in tubers was generally low, it reached up to 0.02% of total soluble protein in comparison with 4% of total protein soluble in potato chloroplasts. In the same year the efficiency of plastid transformation was improved by using of new target regions for insertion of transgenes in the potato plastid genome (Scotti et al., 2011), but this report did not mention about the accumulation of foreign proteins.

Cabbage, as lettuce, represents a plant species with edible leaves containing chloroplasts. The plastid transformation of cabbage was reached by Liu et al. (2007), who expressed the resistant marker gene, *aadA*, and the reporter gene, *uidA*. The study demonstrated a transformation efficiency ranging from 2.7 to 3.3% and a successful accumulation of β-glucuronidase protein in transformed cabbage between 3.2 and 5.2% of total soluble protein. After the development of an efficient plastid transformation in this species, Liu et al. (2008) changed the constructs to express the *cry1Ab* gene targeting to the resistance to *Plutella xylostella* in two cabbage varieties. The *cry1Ab* gene codifies *Bacillus thuringiensis* Cry1Ab delta-endotoxin (Jabeen et al., 2010). The expression of Cry1Ab protein was detected in the range of 4.8–11.1% of total soluble protein in mature leaves of transplastomic plants of the two varieties. This study demonstrated that transplastomic plants displayed significantly higher resistance to *Plutella xylostella* and induces 100% insect mortality after 7 days (Liu et al., 2008).

The only report of carrot plastid transformation was focused on salt tolerance by overexpression of betaine aldehyde dehydrogenase (Kumar et al., 2004). The betaine aldehyde dehydrogenase enzyme activity in carrot transplastomic cells was enhanced eightfold, which accumulated about 50-fold more betaine than cells of control plants. Transplastomic carrot plants grew in the presence of high concentrations of up to 400 mM of NaCl, which is the highest level of salt tolerance reported so far among genetically modified crop plants (Kumar et al., 2004). In this study, it was also observed that the accumulation levels of betaine aldehyde dehydrogenase show a variation dependent on plastid type. The betaine aldehyde dehydrogenase expression reached 74.8% in edible parts (roots), containing chromoplasts, an inferior value compared to leaves (100%), a mainly chloroplasts-containing tissue. This study showed the potential of plastid genome engineering technology to increase salt tolerance in a horticultural crop given that salinity affects drastically and negatively crop productivity and quality (Kumar et al., 2004).

The plastid transformation technology was recently developed for other three horticultural crops as follows: eggplant (Singh et al., 2010; Bansal and Singh, 2014), cauliflower (Nugent et al., 2006) and sugar beet (De Marchis et al., 2009; De Marchis and Bellucci, 2014). These studies did not focus on characteristics of interest for horticulture or agriculture, notwithstanding the plastid genome transformation was developed for them. Although these species have an important economic role in several countries and plastid transformation have the potential to add new traits in order to increase the performance in the field, plastome manipulation have many opportunities in different areas of biotechnology and remains to be done in these species and several others.

Plastid genome sequencing of the target species is an essential tool for correct integration of the transgenes into the plastid genome given that plastid genomes of higher plants are extremely gene-dense and are complexly regulated by operons separated by short intergenic spacer region, which have to be maintained intact given that any disruption can affect the expression of several genes (Shinozaki et al., 1986; Wakasugi et al., 2001; Krech et al., 2012; Bock, 2013). The plastid genome sequencing is also important to identify and characterize endogenous regulatory regions such as promoters, 5′ e 3′ untranslated regions to optimize transgene expression (Ruhlman et al., 2010; Maliga and Bock, 2011; Bock, 2013, 2014). Furthermore, the characterization of endogenous regulatory sequences from plastid genome sequences and transgene expression in edible plant organs containing different plastid types (e.g., leaves, fruits, and tubers) will facilitate the expression of new metabolic pathways and transgenes for the production of healthy nutritional compounds, biopharmaceutical compounds, agriculture useful traits and biomass and raw material for biofuel and chemical industry (Kahlau and Bock, 2008; Valkov et al., 2009; Caroca et al., 2013).

## Concluding Remarks

Plastid genomes are highly conserved with very low rates of substitutions when compared to nuclear genomes. Plastid genes, non-coding regions, RFLP and SSR markers have been frequently used to measure the evolutionary distance at many plant taxonomy levels. This markers are also very helpful for phylogeographical and plant population genetics analyses, as seed and pollen flow studies to the characterize population structure, population differentiation and cytoplasmic diversity (**Figure 1**). However, the limited number of plastid genome sequences for some species, families and genera restrings the quality and efficacy of this kind of analyses. Nowadays, the increasing number of whole plastid genomes are being used for phylogenetic analyses and have proven to be effective tools to resolve evolutionary relationships and genetic diversity or divergence in plant populations, especially at lower taxonomic levels, which limited sequence variation is available. Plastid genome is also an important tool to analyze genetic distance and plant speciation given that it is possible to relate plastid haplotypes with morphological characteristics in natural population as observed in the *Oenothera* genus.

The interesting features of plastid compartment and genome, the exceptional advantages of plastid genome engineering and crescent necessity of horticultural crops for human consumption as food, raw material for industry and cost reduction for production of biopharmaceutical compounds, makes the plastid transformation a potential tool to manipulate different species for industry and food purposes (**Figure 1**). The rapidly growing number of plastid genomes available in the organelle genome resource database can be used to generate high efficient plastid transformation vectors, since sequences of genes, intergenic regions and regulatory elements are crucial information for design of efficient plastid transformation strategies.

Moreover, the improvement of tissue culture system for horticultural crops would help to spread this technology to several species which plastid transformation was not reached at the moment. The regeneration capacity of the tissues is still the bottleneck for a large number of species, given the fact that tobacco has become the model species for plastid transformation due to its high capacity for *in vitro* regeneration.

Due to the high potential and environment-friendly characteristics of plastid engineering, the knowledge acquired during the last two decades about this technology, and the enormous field to be explored in horticultural crops, plastid genomic and transformation constitute a high valuable tool to add new traits and increase the marker value of commercial crops. Moreover, plastid transformation is already safer than nuclear transformation due to exceptionally maternal inheritance of plastids in most angiosperms and lack of dissemination of transgenes via pollen, avoiding contamination of natural germoplasm resources. In addition, horticultural crops can be maintained in closed greenhouse worldwide by using of soil-containing pots or hydroponic systems which can enhance security of transgenic plants, without transgene flux, for several commercial applications.

Finally, plastid genome sequencing is an essential tool for several applications related to plant science. The first knowledge about plastid genome was the starting point to elucidate many processes related to plastid gene function, expression machinery, evolution and transfer of genes to other genetic cellular compartments as mitochondria and the nucleus (**Figure 1**). This gain of knowledge in last three decades, from the first plastid genome sequenced to present day, makes the plastid genome the best studied genetic compartment of the plant cell. The improvement of chloroplast isolation and the evolution of technology of genome sequencing will make plastid genome sequencing routine in many laboratories and will certainly contribute to unveil several unknown questions about plant cell genetic of families/species that no information about plastid genome is available.

## Conflict of Interest Statement

The authors declare that the research was conducted in the absence of any commercial or financial relationships that could be construed as a potential conflict of interest.
